# Velpeau’s Midwifery

**Published:** 1852-05

**Authors:** 


VELPEAU'S MIDWIFERY.

This is the fourth American edition of Velpeau's midwifery, translated by Prof. Meigs, embracing additions by the author, and a history of the obstetric art, which add much to the interest and value of this popular treatise. The first edition appeared more than twenty years since, and the fact that another is now demanded, after the lapse of so much time, is the best proof of the substantial excellence of the work. Velpeau is one of the masters of our profession. The remark of Dr. Meigs, that he "stands scarcely second to any physician in Europe," remains still as true as when it was made many years ago. This edition of his treatise is published by Lindsay and Blakiston, who have bestowed ample care upon its mechanical execution. Prefixed to it is a very full table of contents, which for reference will be found of the utmost convenience. The illustrations are numerous and truthful. In a word, it is what it professes to be, a *complete* treatise on midwifery.

